# Orthopedics on the Rise: Billing Trends Reveal Changing Roles in Spine Surgery

**DOI:** 10.7759/cureus.96836

**Published:** 2025-11-14

**Authors:** Graham A Branscom, Leilei Hao, Brendan F Judy, Rebecca Carpenter, David S Casper, John Y.K. Lee

**Affiliations:** 1 Neurosurgery, University of Pennsylvania, Philadelphia, USA; 2 Orthopedic Surgery, University of Pennsylvania, Philadelphia, USA; 3 Neurosurgery, Hospital of the University of Pennsylvania, Philadelphia, USA

**Keywords:** anterior lumbar interbody fusion (alif), cervical spine procedures, medicare data, physician specialty, procedure volumes, spinal deformity correction

## Abstract

Background

Spine surgery is a shared domain between neurosurgery and orthopedic surgery, yet the exact differences between the two specialties have not been fully elucidated. The aim of this study is to investigate the differences in procedural volume and the recent trends indicating shifting roles of these two specialties in spine surgery. Understanding these trends is critical for workforce planning, training, and interdisciplinary collaboration.

Methods

This study analyzed the publicly available Medicare Part B dataset containing procedure-level data from 2013 to 2022. After filtering, we analyzed data from 1,695 and 1,531 spine neurosurgeons and orthopedic surgeons, respectively.

Results

Spine neurosurgeons have a higher volume of new patient office visits than orthopedic spine surgeons (median per surgeon from 2013-22: 1015 (IQR: 793.5) vs. 948 (807.8), respectively) (*p*<0.01). However, orthopaedic spine surgeons have a higher volume of established patient visits (3736 [3561.5] vs. 1894.5 [1749.3]) (*p*<0.0001) and overall spinal procedure volumes (576 [562] vs. 480 [468.5]) (*p*<0.0001). Additionally, the majority of all spinal deformity and ALIF procedures are performed by orthopaedic surgeons (61.8% and 68.2%, respectively), but neurosurgeons are performing an increasing proportion of these procedures over time (*p*<0.0001). Conversely, although the majority (66.1%) of all cervical procedures are performed by neurosurgeons, orthopedic surgeons are performing an increasing proportion of these procedures (*p*<0.0001).

Conclusions

Although orthopedic spine surgeons are performing a higher volume of procedures than spine neurosurgeons, both specialties are slowly conducting a greater proportion of certain types of procedures that have been historically dominated by other specialties.

## Introduction

Spine surgery has long been a shared domain between neurosurgery and orthopedic surgery. While the types of procedures completed by each specialty are largely similar, previous research has investigated some of the differences in volume and outcome between the two specialties. Orthopedic surgeons were found to perform more spinal deformity procedures and kyphoplasty, yet neurosurgeons perform more one- to six-level arthrodesis and anterior cervical discectomy and fusion [1-5].

The current landscape of literature discussing the differences between spinal neurosurgery and orthopedic surgery is limited. Several studies have investigated the differences in complication rates between neurosurgery and orthopedic surgery following spinal procedures and report little to no differences in complication rates across both specialties [3,6-16]. Although these studies provide definitive outcome measurements, they often report data on the individual patient level rather than the surgeon level. Thus, it may be difficult to draw conclusions from these studies about the overall population of neurosurgery versus orthopedic spine surgeons. Much of the remaining literature discussing the procedural volume difference between neurosurgery and orthopedic spine surgery pertains to residency training [17-19]. For instance, graduating neurosurgery residents had 6.8 times higher spinal volumes than orthopedic surgery residents, and the rate of neurosurgery residents’ spinal case volumes has been increasing [17]. However, these studies’ findings are only informative for understanding differences in surgical training and cannot be easily extrapolated to overall specialty-wide differences. The remaining area of literature discusses differences in reimbursement rates [20-22]. On average, neurosurgeons receive higher reimbursement rates than orthopedic surgeons for spinal procedures [20]. However, these studies do not provide insight into surgical performance in terms of volume or complication rates. The few studies that have analyzed Medicare volume data for spine surgery do not rely on the most recent data and are limited to only certain procedures [23].

Given this state of the literature, there is some understanding of the differences between neurosurgery and orthopedic spine surgery, but there is a current lack of studies that comprehensively compare the two specialties’ volumes across all spinal procedures using an updated data source. This study utilizes a Medicare Part B dataset of all spine neurosurgeons and orthopedic surgeons in the US to compare surgical volumes. Our objective is to identify specific procedure types that are similar and different across the two specialties and to elucidate how these trends are actively changing over time. We hope that this novel and exhaustive approach will be clinically useful and inform the practice of spine surgeons across both specialties.

## Materials and methods

Data source

We used a publicly available dataset from the Center for Medicare and Medicaid Services called “Medicare Physician and Other Practitioners by Provider and Service” (2022) [24]. The dataset contains information on services provided to the Original Medicare Part B beneficiaries. Each row in the data represents one unique entry for a unique national provider identifier (NPI), Healthcare Common Procedure Coding System (HCPCS) code, and place of service. If one NPI billed the same HCPCS code multiple times at the same place of service, this is one entry in the dataset. The dataset contains information from 2013 to 2022 (inclusive).

Data filtering and analysis

**Figure 1:**
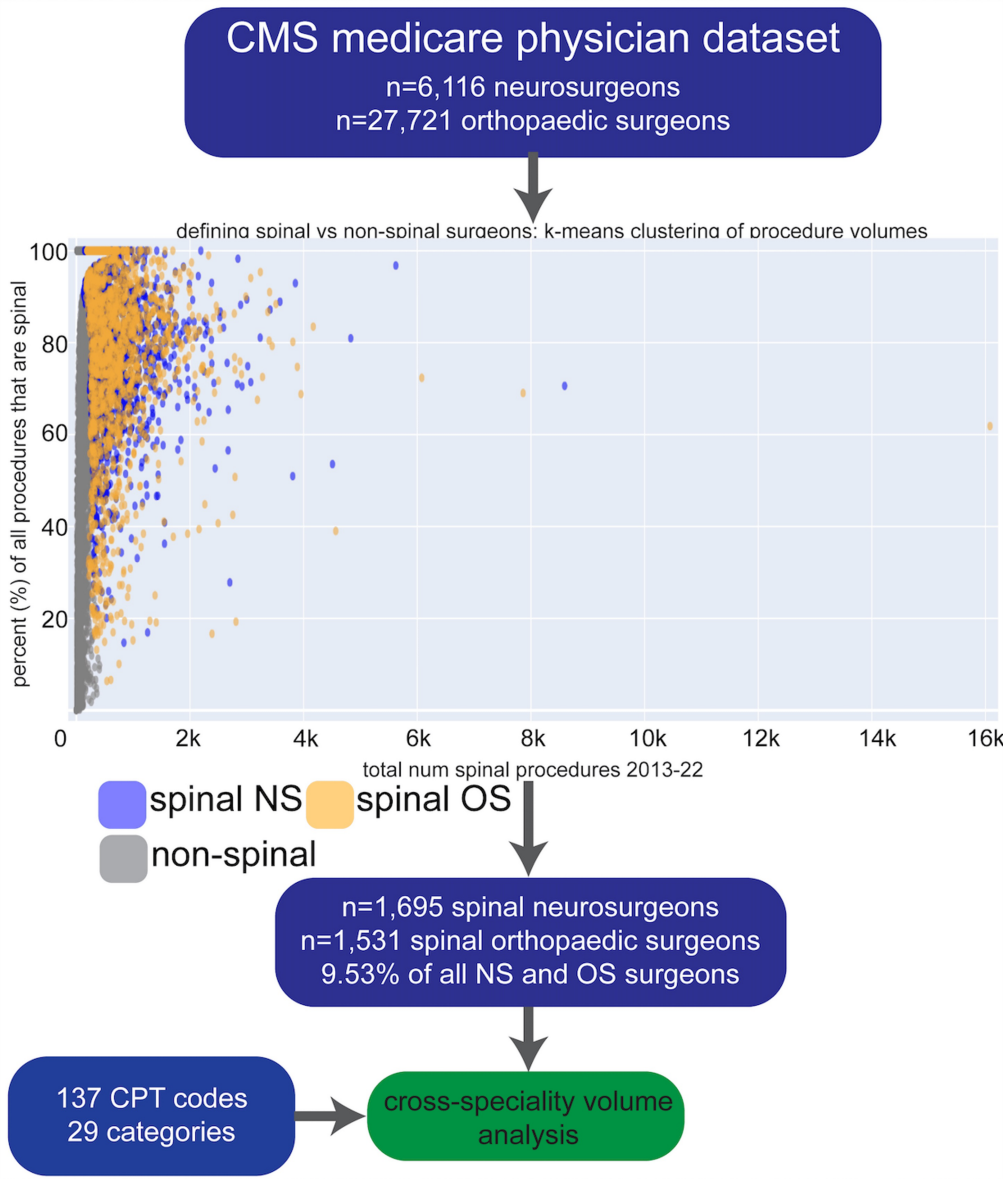
Methodology of defining spine surgeons We utilized the publicly available Center for Medicare and Medicaid Services (CMS) Medicare Part B dataset of n=6,116 neurosurgeons and n=27,721 orthopedic surgeons. For each surgeon, we graphed the total number of spinal procedures from 2013 to 2022 versus the percentage of all their surgical procedures that are spinal. We used k=15 clusters and considered spine surgeons to be those in the 14-15 clusters with the highest values for these two metrics. This resulted in n=1,695 spine neurosurgeons and n=1,531 orthopedic spine surgeons, representing 9.53% of the total original number of surgeons. We compared procedural volumes across 137 Current Procedural Terminology (CPT) codes split among 29 categories of procedures.

Our data filtering scheme is described in Figure 1. We first filtered the dataset for only NPIs whose specialty is neurosurgery or orthopedic surgery (n=6,116 neurosurgeons, n=27,721 orthopedic surgeons). We then filtered to only include spine surgeons by running k-means clustering (seed=3) on plots of spinal procedure volumes vs. percentage of all surgical procedures that are spinal. We made separate plots for all the neurosurgeons and orthopedic surgeons. K-means clustering is a machine learning technique that allows us to find natural clusterings in the dataset as opposed to coming up with arbitrary thresholds. We used k=20 clusters and considered spine surgeons to be those in the 14-15 clusters with the highest values for these two metrics. This resulted in n=1,695 spine neurosurgeons and n=1,531 orthopedic spine surgeons, representing 9.53% of the total original number of surgeons. Spinal procedures were any procedure with a primary spine surgery Current Procedural Terminology (CPT) code. We consulted the Departments of Neurosurgery and Orthopaedic Surgery at our institution to form a comprehensive list of these spine procedure CPT codes (Table 1). We included primary CPT codes that represent the main surgical procedure, whereas we excluded add-on codes such as 63048 (laminectomy on an additional vertebral segment) and 61783 (spinal stereotactic navigational planning) (Table 2). We also excluded all spinal tap CPT codes from the primary procedures. CPT codes were grouped according to broad categories that we defined. In total, there were 137 CPT codes across 29 categories. Each CPT code was assigned exactly one category. If applicable, CPT codes were assigned to the one specific region of the spinal column that they are relevant to (lumbar, thoracic, cervical, etc.). Although we included 29 categories, we only reported on the results that we found most statistically significant and interesting to the field. Throughout the manuscript, volumes are reported as median (interquartile range [IQR]).

**Table 1:** Spinal CPT codes for primary procedures CPT: Current Procedural Terminology

Category	Anatomical location	CPT
anterior lumbar interbody fusion	lumbar	22558
anterior cervical discectomy, decompression, and fusion	cervical	22551
		63075
anterior/thoracic fusion without decompression	cervical	22554
	thoracic	22556
laminectomy without facetectomy, foraminotomy	cervical	63001
		63015
	thoracic	63003
		63016
	lumbar	63005
laminectomy with facetectomy, foraminotomy	cervical	63045
	thoracic	63046
	lumbar	63047
discectomy	cervical	63020
		63040
	thoracic	63077
	lumbar	63030
		63042
other posterior lumbar decompression/discectomy procedures	thoracic	63055
		63064
	lumbar	63012
		63056
		62287
posterior fusion	cervical	22600
		22595
		22590
	thoracic	22610
		22532
	lumbar	22630
		22633
		22612
		22533
	N/A	22800
		22802
		22804
posterior spine - new technology	lumbar	22867
		22869
exloration of fusion and hardware removal/reinsertion	N/A	22830
		22849
		22850
		22852
		22855
spine fracture	cervical	22326
	thoracic	22327
	lumbar	22318
		22319
		22325
	N/A	20660
		20661
		22310
		22315
corpectomy	cervical	63081
		63300
		63304
	thoracic	63101
		63305
		63306
		63301
		63302
		63085
	thoracolumbar	63087
		63090
		63303
		63307
		63102
total disc arthroplasty	cervical	22856
		0375T
	lumbar	22857
sacroiliac join stabilization/arthrodesis	sacral	27279
		27280
vertebroplasty	cerviothoracic	22510
	lumbosacral	22511
vertebral augmentation	thoracic	22513
	lumbosacral	22514
spinal deformity	cervical	22220
		22210
	thoracic	22222
		22212
	thoracolumbar	22206
	lumbar	22224
		22214
		22207
	N/A	22808
		22810
		22812
laminectomy for spinal tumors	cervical	63275
		63280
		63285
	thoracic	63276
		63281
		63286
	thoracolumbar	63287
	lumbar	63277
		63282
	sacral	63278
		63283
	coccygeal	49215
	N/A	63290
laminectomy for non-neoplasms (hematoma/abscess)	cervical	63265
		63270
	thoracic	63266
		63271
	lumbar	63267
		63272
	sacral	63268
		63273
spinal abscess not requiring laminectomy	cerviothoracic	22010
	lumbosacral	22015
laminectomy for spinal cord arteriovenous malformations	cervical	63250
	thoracic	63251
	thoracolumbar	63252
spinal dura repair	N/A	63707
		63709
		63710
stereotactic radiosurgery (spinal)	N/A	63620
spinal neurotransmitters	N/A	63650
		63655
		63685
		63661
		63662
		63663
		63664
		63688
spinal pumps	N/A	62350
		62351
		62360
		62361
		62362
		62355
		62365
irrigation and debridement	N/A	10140
		97597
		97605
osteophyte resection	cervical	22110
laminoplasty	N/A	63050
		63051
laminotomy	N/A	64727

**Table 2:** Add-on spinal procedure CPT codes CPT: Current Procedural Terminology

Category	CPT
anterior lumbar interbody fusion	22585
	22840
	22842
	22843
	22844
	22848
	22853
	22854
	22859
	22845
	22846
	22847
	20930
	20931
	20936
	20937
	20938
	20939
	61783
anterior cervical discectomy, decompression, and fusion	22552
	22853
	22854
	22859
	22845
	22846
	22847
	20930
	20931
	20936
	20937
	20938
	20939
	61783
laminectomy without facetectomy, foraminotomy	63048
	63052
	63053
disectomy	63035
	63043
	63044
other posterior lumbar decompression/discectomy procedures	63057
	63057
	63066
posterior fusion	22614
	63052
	63053
	22632
	22634
	22534
	22840
	22842
	22843
	22844
	22848
	22853
	22854
	22859
	20930
	20931
	20936
	20937
	20938
	20939
	61783
posterior spine - new technology	22868
	22870
spine fracture	22328
corpectomy	63082
	63086
	63088
	63091
	63308
	63103
total disc arthroplasty	22858
	0163T
vertebroplasty	22512
vertebral augmentation	22515
spinal deformity	22226
	22216
	22208
laminectomy for spinal tumors	69990
	63295
laminectomy for non-neoplasms (hematoma/abscess)	69990
	63295
laminectomy for spinal cord arteriovenous malformations	69990
stereotactic radiosurgery (spinal)	63621

Additionally, we conducted some analyses that compared spinal procedure volumes to total surgical volumes. Total surgical volumes were found by removing all CPT codes related to surgical add-ons, in-office visits, in-patient medical care, injections, laboratory tests, and imaging.

When calculating the changes in the proportion of NS or OS that conduct a certain spinal procedure category over time, we ran a logistic regression model and found the beta and p-value associated with this regression. We used this as a way of assessing whether one specialty is performing an increasing or decreasing proportion of procedures within that category.

Statistical tests

Data distributions were determined by the Shapiro-Wilk test to be non-normal in all our analyses. Therefore, we relied on non-parametric tests downstream. We used the Wilcoxon rank-sum test to compare volumes between neurosurgery and orthopaedic surgery. Lastly, we used the Wald test for our logistic regression models for the time course data. For all statistical tests, we used a significance cutoff of p<0.05.

Software

All analyses were completed using Python (v3.11.12) in the Google Colab environment. Data cleaning and restructuring were done using the Python package Pandas (v2.2.2). We used scikit-learn (v1.6.1) for k-means clustering to determine spinal vs. non-spine surgeons. Figures were generated using Plotly (v5.24.1), and statistics were run using Scipy (v1.15.3). Figures were edited and rendered using Adobe Illustrator (v29.5.1).

## Results

Distribution of spinal procedures among spine surgeons

**Figure 2:**
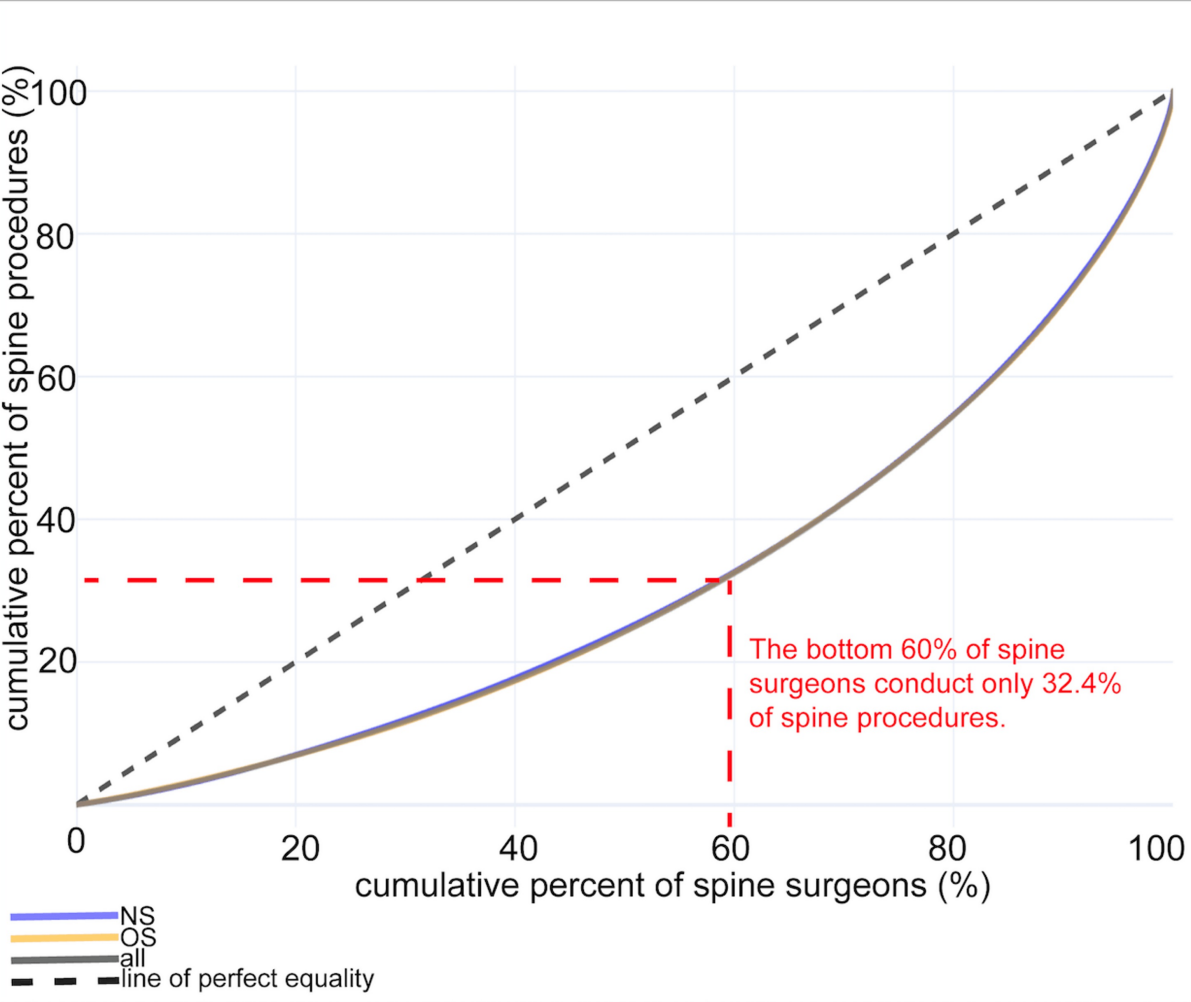
A minority of spine surgeons perform the majority of procedures Of the n=1,695 spine neurosurgeons (NS) and n=1,531 orthopedic spine surgeons (OS), we graphed the distribution of spinal procedures in a Lorenz curve, which is used to identify inequalities in distributions. We found that the bottom 60% of spine surgeons conduct just 32.4% of procedures, i.e., the top 40% of spine surgeons conduct 67.6% of procedures. Note that the three lines are overlapping.

We graphed the relationship between the distribution of spine surgeons and the number of procedures they have completed in a Lorenz curve, which is used to visualize inequalities in distributions [25]. We found that the bottom 60% of spine surgeons perform only 32.4% of spine procedures, i.e., the top 40% of spine surgeons perform 67.6% of spine procedures (Figure 2).

In-office visits and spinal procedures

**Figure 3:**
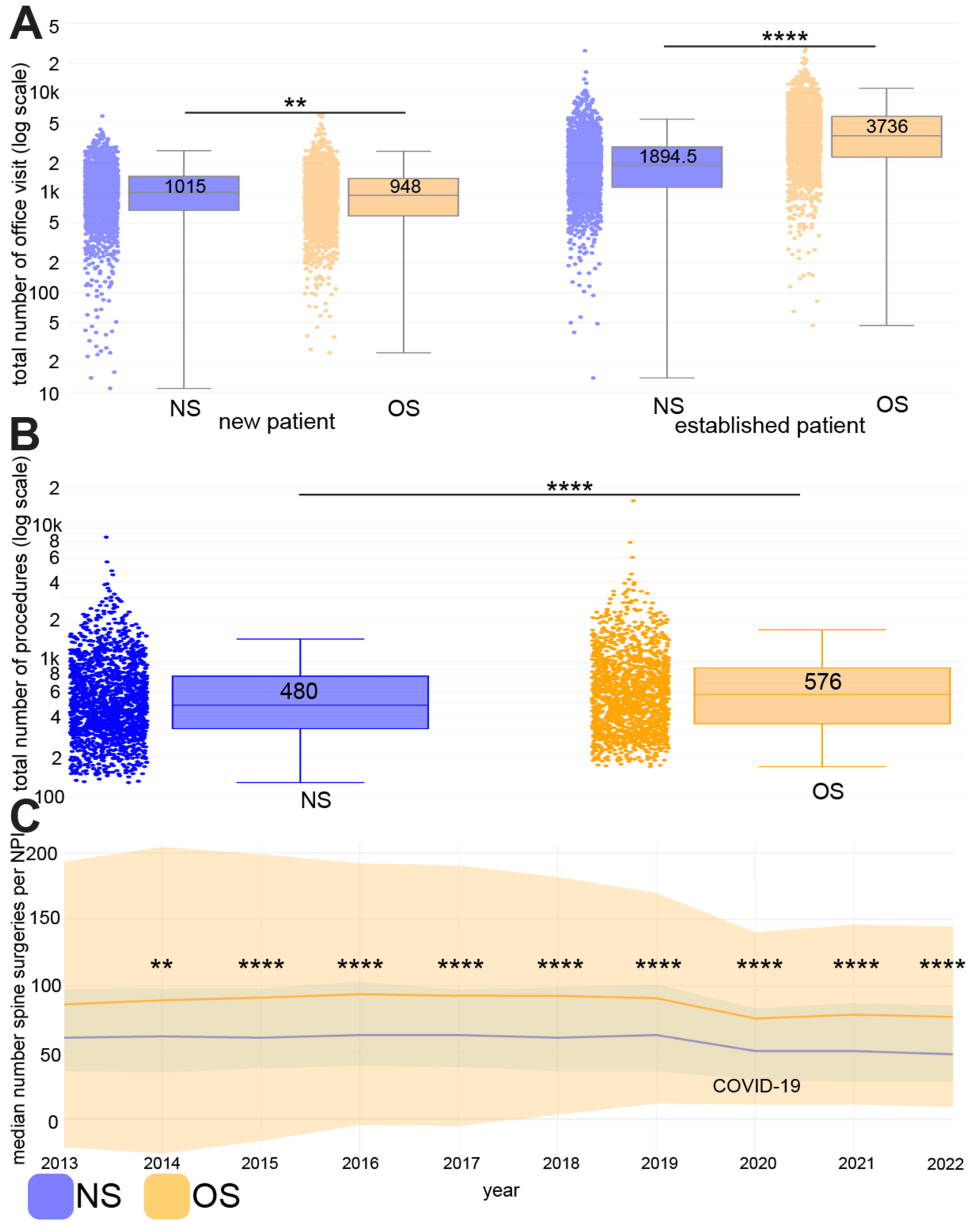
Spine neurosurgeons have more visits with new patients, but orthopedic spine surgeons have higher procedure volumes A) Office visit volumes separated by patient type. Each surgeon is a separate point on the graph, with n=1,695 spine neurosurgeons (NS) and n=1,531 orthopedic spine surgeons (OS). The y-axis represents the total number of office visits from 2013 to 2022 and is on a log scale. B) Spinal procedure volumes. Each surgeon is a separate point on the graph, with n=1,695 spine neurosurgeons (NS) and n=1,531 orthopedic spine surgeons (OS). The y-axis represents the total number of primary spinal procedure CPT codes billed for that surgeon from 2013 to 2022 and is on a log scale. C) Spinal procedure volumes over time from 2013 to 2022. The y-axis represents the median number of spine surgeries per national provider identifier (NPI) (surgeon). We used the Wilcoxon rank-sum test to compare volumes between neurosurgery and orthopedic surgery. ** p<0.01 **** p<0.0001

We found that spine neurosurgeons have more new patient visits (median per surgeon from 2013-22: 1015 [IQR: 793.5]) than orthopedic spine surgeons (948 [807.8]) (p<0.01). However, spine neurosurgeons have much less established patient visits (median per surgeon from 2013-22: 1894.5 [1749.3]) than orthopedic spine surgeons (3736 [3561.5]) (p<0.0001) (Figure 3A).

In terms of spinal procedures, orthopedic spine surgeons perform a higher volume of spinal procedures (median per surgeon from 2013-22: 576 [562]) than spine neurosurgeons (480 [468.5]) (p<0.0001) (Figure 3B). This trend holds true each year from 2014 to 2022 (p<0.01). As a side note, the overall spinal procedure volumes have remained steady from 2013 to 2019 and saw a significant drop in 2020, likely due to COVID-19 [25]. However, volumes have risen slightly in the two years afterward (Figure 3C).

**Figure 4:**
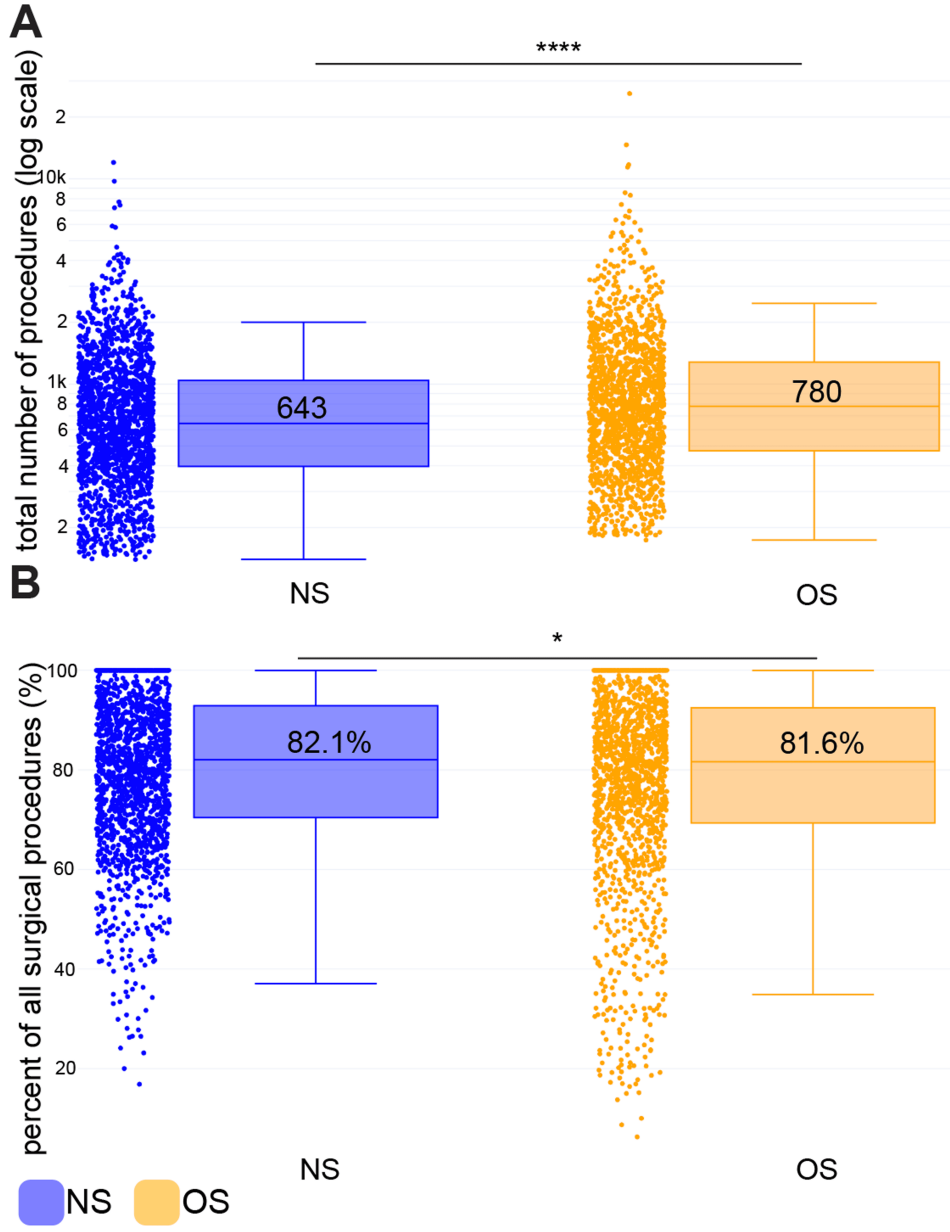
Orthopedic spine surgeons have higher overall surgical volumes (outside of just spine) than spine neurosurgeons, yet the percentage of procedures that are spinal is relatively similar across both specialties A) All surgical procedure volumes (including spine and other types of procedures like knee surgery) among n=1,695 spine neurosurgeons (NS) and n=1,531 orthopedic spine surgeons (OS). The y-axis represents the total number of primary surgical procedures billed for a surgeon from 2013 to 2022 and is log scale. B) The percentage of all surgical procedures that are spinal among spine surgeons. This number represents the total number of primary spinal procedures divided by the total number of primary surgical procedures from 2013 to 2022. We used the Wilcoxon rank-sum test to compare volumes between neurosurgery and orthopedic surgery. * p<0.05 **** p<0.0001

We also examined overall surgical volumes across all procedure types, not just spine, to better understand volume differences. From 2013 to 2022, orthopaedic spine surgeons had significantly higher total procedure volumes per surgeon (median: 780 [806]) compared to spine neurosurgeons (median: 643 [645], p < 0.0001) (Figure 4A). However, the proportion of each surgeon’s total procedures that were spine-related was relatively similar between specialties (neurosurgery: 82.1% [22.5%], orthopedics: 81.6% [23.1%]) (Figure 4B). These findings suggest that orthopedic spine surgeons perform more procedures overall, but this is not due to performing more non-spine surgeries. Instead, the higher overall volumes appear to be driven primarily by greater numbers of spine procedures.

Changes within categories of spine surgery

**Figure 5:**
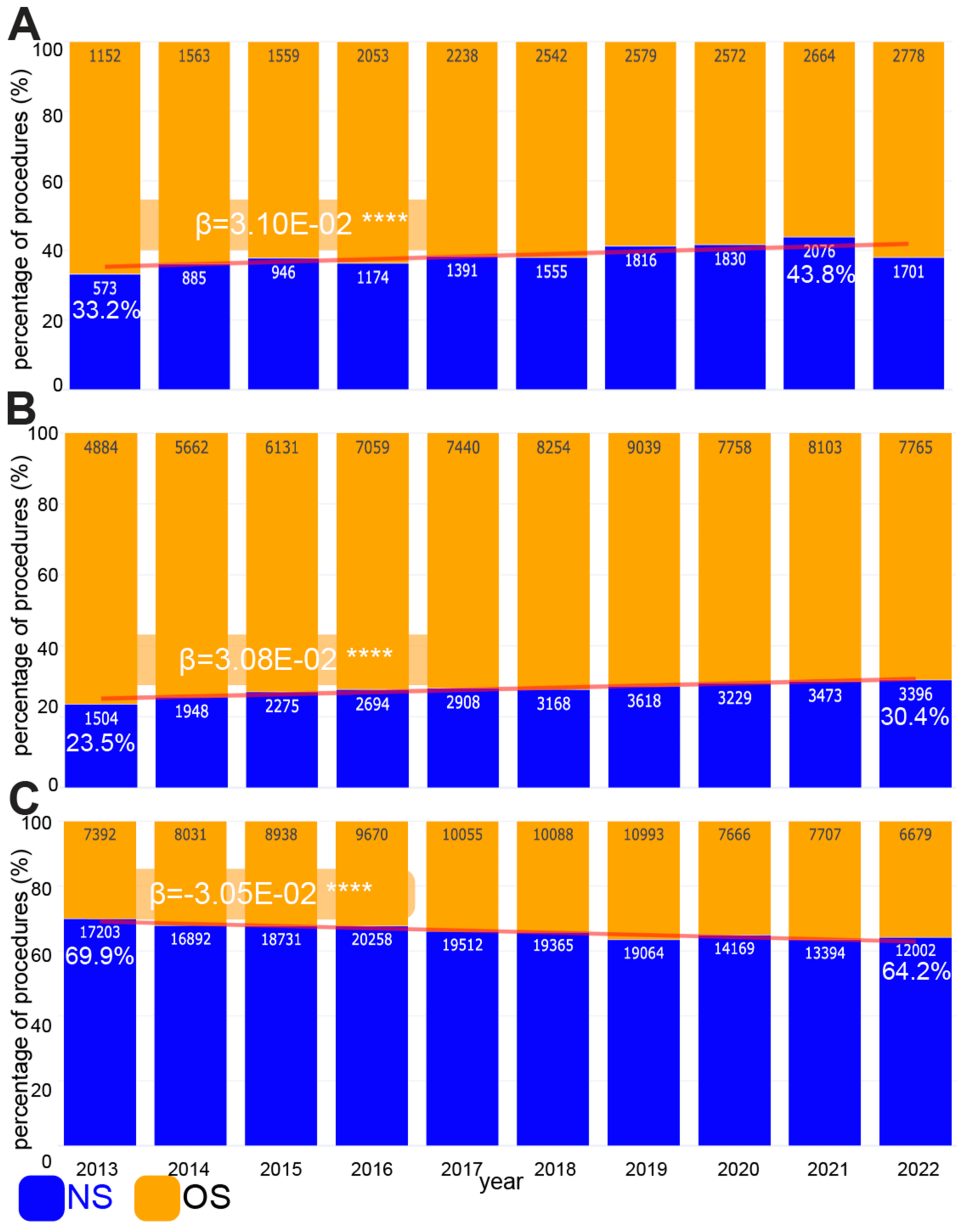
Spine neurosurgeons are performing an increasing proportion of deformity and ALIF procedures and a decreasing proportion of cervical procedures We show the proportion of procedures from 2013 to 2022 performed by all n=1,695 spine neurosurgeons (NS) and n=1,531 orthopedic spine surgeons (OS). The number within each bar shows the total number of procedures performed by that specialty within that year. The percentage performed by neurosurgery in 2013 and 2021/22 is also shown. We used a logistic repression model on these proportions and reported the beta value to show significant changes over time. We used the Wald test for our logistic regression models. A) Spinal deformity procedures B) Anterior lumbar interbody fusion (ALIF) procedures C) Cervical procedures **** p<0.0001

Lastly, we wanted to identify whether one specialty is conducting an increasing proportion of a category of spinal procedures that the other specialty traditionally dominates. We found that although 61.8% of spinal deformity procedures are conducted by orthopedic spine surgeons, spine neurosurgeons are performing an increasing proportion of these procedures, from 33.2% in 2013 to 43.8% in 2021. This is a significant increase over time (β=3.10E-02, p<0.0001) (Figure 5A). Similarly, orthopedic spine surgeons have historically performed the majority (68.2%) of anterior lumbar interbody fusion (ALIF) procedures. However, spine neurosurgeons are performing an increasing proportion of ALIF procedures over time, from 23.5% in 2013 to 30.4% in 2022. This is also a significant increase over time (β=3.08E-02, p<0.0001) (Figure 5B).

Conversely, spine neurosurgeons perform 66.1% procedures that are located within the cervical spine. However, spinal orthopedic surgeons are performing an increasing proportion of cervical procedures over time, from 30.1% in 2013 to 35.8% in 2022 (β=-3.05E-02, p<0.0001) (Figure 5C).

## Discussion

In summary, we conducted an analysis of 10 years of spine surgery Medicare data. We first developed a novel technique to define spine surgeons from non-spine surgeons based on total spinal procedure volumes and the percentage of all procedures that are spinal, using the k-means clustering machine learning technique. Among these surgeons, we found that a minority of neurosurgery and orthopedic spine surgeons perform the majority of the procedures. Next, we found that although spine neurosurgeons have more new patient visits, orthopedic spine surgeons have more established patient visits and spinal procedures. Lastly, we showed that although orthopedic spine surgeons have historically performed the majority of spinal deformity and ALIF procedures, the proportion of spine neurosurgeons performing those procedures is slowly increasing. Conversely, spine neurosurgeons have performed the majority of cervical procedures, although the proportion performed by orthopedic spine surgeons is slowly increasing.

Comparison to prior literature

In terms of the distribution of spinal procedures among spine surgeons, other studies have found similar results. An analysis of case volumes from 644 spine surgeons in the British Spine Registry found that the top 37.7% of surgeons performed 80% of all operations [26]. While our results were less extreme (40% of surgeons performing 67.7% of procedures), the consistent presence of some level of volume inequality across international datasets highlights a potential structural feature of spine surgery practice.

Although no studies have directly compared overall spinal procedure volume differences between neurosurgery and orthopedic surgery, other studies have compared differences within individual procedures. A US study of 12,929 patients who underwent adult spinal deformity procedures found that orthopedic surgeons performed most deformity procedures (64.6%), and the proportion performed by neurosurgeons has increased from 24.4% in 2010 to 35.2% in 2019 (p<0.001) [1]. Our results reflect very similar statistics and trends. Although that study was conducted using a different dataset that covered a different patient population, it shows that our results may extend to other patient populations across the country.

An assessment of spine surgeons in the American College of Surgeons National Surgical Quality Improvement Program (ACS-NSQIP) database found that orthopedic surgeons perform 55.1% of lumbar fusions, a similar result to our own for ALIF [4]. However, no studies have reported how this proportion has changed over time. Instead, studies have only shown that the overall ALIF volumes are increasing over time [21]. Similarly, for cervical procedures, other studies have reported that neurosurgeons perform the majority of cases. Specifically, studies of just anterior cervical decompression and fusion (ACDF) cases in the American College of Surgeons National Surgical Quality Improvement Program (ACS-NSQIP) database found that neurosurgeons performed 74% of ACDF procedures [2,3].

Strengths

This study used a robust dataset of all the spine surgeons in the US who treat the Medicare population. We included data across 10 years of their practice, giving us a comprehensive understanding of both overall and temporal differences across both specialties. The breadth of our data can increase confidence in the conclusions that we draw from it. Additionally, we used a methodical approach for defining spine surgeons from non-spine surgeons. Given that surgeons can practice across multiple types of procedures, it is important to logically define this boundary.

Limitations

A major limitation of our study is that we only assessed differences in volumes; we did not directly assess procedure outcomes. However, several studies have shown that higher spinal procedure volumes are associated with better patient outcomes, such as lower post-operative morbidity and mortality [27-29]. Thus, our assessment of procedural volumes may serve as a meaningful proxy for outcomes in this context.

Another limitation is that the publicly available Medicare Part B dataset that we analyzed only includes data up to 2022. As additional years of data are released, longitudinal analyses will be critical to determine whether these patterns persist or evolve.

Implications

First, since prior literature has shown that higher spine procedure volumes are associated with improved outcomes [27-29], understanding the differences between neurosurgeon and orthopaedic spine surgeons may have important implications for patient referrals. Depending on the specific condition a patient presents with, one specialty may be better suited for referral than another. This may improve patient outcomes and improve the efficiency of healthcare delivery. Additionally, understanding these differences may help inform orthopedic surgery and neurosurgery training programs. Understanding the highest volume procedures within each specialty may help improve trainees’ preparation for the conditions they may treat more frequently throughout their surgical careers.

Lastly, we hope the methodology used to conduct this study will guide future research. Due to the mixed nature of surgical practice, it is sometimes difficult to precisely define one subspeciality versus another. There was no clear boundary between spine and non-spine surgeons among all orthopedic surgeons and neurosurgeons in this study. We developed a novel framework of defining surgeons’ subspecialties based on conducting the k-means clustering machine learning framework on a distribution of their procedure volumes. Additionally, our approach was novel in terms of drawing volume comparisons across the two specialties using a comprehensive Medicare dataset. We hope that researchers conducting similar work can use this framework to delineate subspecialties from one another.

## Conclusions

Overall, our results show that orthopedic spine surgeons perform a higher volume of procedures than spine neurosurgeons. However, regarding some categories of procedures like spinal deformity, ALIF, and cervical procedures, this landscape is actively changing. While our study is limited to volume-based analyses, it still provides valuable insights into recent changes in spine surgery. Future research should continue to monitor these patterns and identify changing roles between the two specialties.
